# Vitamin D Deficiency and Leisure Time Activities in the Elderly: Are All Pastimes the Same?

**DOI:** 10.1371/journal.pone.0094805

**Published:** 2014-04-10

**Authors:** Marina De Rui, Elena Debora Toffanello, Nicola Veronese, Sabina Zambon, Francesco Bolzetta, Leonardo Sartori, Estella Musacchio, Maria Chiara Corti, Giovannella Baggio, Gaetano Crepaldi, Egle Perissinotto, Enzo Manzato, Giuseppe Sergi

**Affiliations:** 1 Department of Medicine, Geriatric Division, University of Padova, Padova, Italy; 2 Department of Medical and Surgical Sciences, University of Padova, Padova, Italy; 3 Azienda Unità Locale Socio Sanitaria n. 16, Padova, Italy; 4 Department of Medicine, Internal Medicine Division, Azienda Ospedaliera, Padova, Italy; 5 National Research Council, Aging Branch, Institute of Neuroscience, Padova, Italy; 6 Department of Cardiac, Thoracic and Vascular Sciences, Biostatistics, Epidemiology and Public Health Unit, University of Padova, Padova, Italy; IPO, Inst Port Oncology, Portugal

## Abstract

**Background:**

Optimal vitamin D status is important for overall health and well-being, particularly in the elderly. Although vitamin D synthesis in the skin declines with age, exposure to sunlight still seems to help older-aged adults to achieve adequate serum 25-hydroxyvitamin D (25OHD) levels. Elderly people would therefore benefit from outdoor leisure activities, but the effects of different types of pastime on serum 25OHD levels have yet to be thoroughly investigated.

**Aims:**

To assess the association of different pastimes with 25OHD deficiency in elderly subjects.

**Methods:**

A sample of 2,349 community-dwelling elderly individuals (1,389 females and 960 males) enrolled in the *Progetto Veneto Anziani* was analyzed. Brisk walking, cycling, gardening and fishing were classed as outdoor activities, and dancing and gym workouts as indoor pastimes. Any activities undertaken for at least 1 hour/week during the previous month were considered as being practiced regularly. Logistic regression models were used to estimate the association between different pastimes and 25OHD deficiency.

**Results:**

Serum 25OHD levels were significantly higher in individuals who engaged in outdoor pastimes (+25% in women, +27.7% in men) compared to those who did not. In particular, subjects regularly practicing gardening or cycling had higher serum 25OHD levels than those who did not, whereas 25OHD levels differed little between subjects who did or did not undertake indoor activities. Among the outdoor pastimes considered, logistic regression analysis confirmed a lower likelihood of vitamin D deficiency (25OHD<50 nmol/L) for cyclists (OR 0.51, 95% CI 0.37–0.69 in women; OR 0.50, 95% CI 0.29–0.87 in men) and gardeners (OR 0.62, 95% CI 0.47–0.83 in women; OR 0.46, 95% CI 0.26–0.80), but not for brisk walkers.

**Conclusions:**

Regular cycling and gardening reduce the likelihood of inadequate vitamin D status in Caucasian elderly people, irrespective of their age, BMI and comorbidities, and of the season of the year.

## Introduction

The role of vitamin D in bone health and muscle function has long been recognized, and an increasing body of evidence suggests that inadequate vitamin D levels may contribute not only to osteoporosis, but also to cardiovascular disorders, type 2 diabetes and cancer [1]–[3]. Vitamin D deficiency is a frequent finding among older adults, reaching a prevalence beyond 50% in various epidemiological studies [4], [5]. Several factors may account for this hypovitaminosis D *epidemic* among elderly people, such as a reduced dietary vitamin D intake or an increased vitamin D storage in adipose tissue [6], but the main culprit is thought to be the age-related decrease in 7-dehydrocholesterol (7-DHC) content in the epidermis, which means an impoverished substrate for vitamin D production [7]. Recommendations concerning exposure to sunlight have consequently been overshadowed by the prescription of oral supplements, which are generally the preferred method for preventing or correcting hypovitaminosis D. Despite the age-related decline in vitamin D production by the skin, there is some evidence that older adults can still maintain adequate serum vitamin D levels by taking advantage of exposure to ultraviolet light (UV) [8]. Since older adults have more leisure time and therefore more opportunities to spend time outdoors, promoting regular activities that involve their exposure to sunlight could contribute to preventing hypovitaminosis D.

To the best of our knowledge, few studies [9]–[11] have investigated the relationship between physical activity and vitamin D status in the elderly, and none have considered the effects of different outdoor pastimes on serum 25OHD levels. Identifying the activities associated with a lower probability of hypovitaminosis D in elderly subjects might have important implications for public health.

We hypothesized that, for older adults, regular outdoor leisure-time activities entailing exposure to sunlight, such as brisk walking, gardening, cycling, and so on, might not have the same effect on serum 25OHD levels. The aim of the present study was thus to assess the association of different regular physical activities with vitamin D deficiency in a population-based sample of healthy community-dwelling elderly subjects.

## Methods

### Ethics approval

The local Ethical Committees of Padua University and of the Local Health Units (ULSS) n. 15 and n. 18 of the Veneto Region approved the study protocol, and participants gave their written informed consent.

### Data source and subjects

The data considered in this analysis were drawn from the *Progetto Veneto Anziani* (Pro.V.A.), an observational cohort study on the Italian population aged ≥65 years living in two geographical areas in the north-east of Italy (Camposampiero and Rovigo). The study population included 3,099 age- and sex-stratified Caucasian community-dwelling participants (1,245 men and 1,854 women) randomly selected between 1995 and 1997 using a multistage stratified method designed to keep the male-to-female ratio at 2:3 and to oversample the oldest age group. Sampling procedures and data collection methods have been described in detail elsewhere [12].

Participants were examined at city hospitals by trained physicians and nurses. Health status was ascertained by integrating information obtained from a physical examination and a review of the medical records. For the analyses conducted in the present study, participants lacking serum 25OHD test results (n = 272) and those in wheelchairs or unable to walk (n = 89), or with leg and/or arm amputations (n = 23) were excluded. Cases diagnosed with primitive hyperparathyroidism (n = 17) or moderate-to-severe renal failure (n = 11) (defined as a glomerular filtration rate <30 ml/minute) were also excluded. Participants' physical performance status and aerobic capacity were measured using the standardized 6-minute walking test (6MWT) [13], and those unable to walk at their usual pace for six minutes (n = 338) were excluded. The final sample consisted of 2,349 well-performing and self-sufficient individuals with a complete set of data on their physical activities and comorbidities.

### Clinical and laboratory data

Information was collected on participants' formal education, smoking and regular exercising habits during an in-person interview. Educational level (the total number of years of school attended) was dichotomized as ≤5 versus >5 years of schooling. Smoking habits were classified as “never/former” (for at least a year in the past) versus “current” smokers. Physical activities were grouped into two categories, i.e. outdoor activities (brisk walking, cycling, gardening, and fishing), and indoor activities (dancing, exercising at the gym). Participants were asked to report how many hours a week they had spent on each of the above-mentioned pastimes in the previous month. An activity was considered regular if it had been practiced for more than 1 hour a week during the previous month.

Any medical conditions were identified by board-certified physicians involved in the study, who examined all the clinical information collected on each participant, including clinical history, self-reported symptoms (using standardized questionnaires), medical and hospital records, blood tests, and a physical examination. The prior major diseases considered were any of the following: cardiovascular diseases (CVD: congestive heart failure, angina and myocardial infarction, stroke, and peripheral artery disease), diabetes, chronic obstructive pulmonary diseases (COPD), cancer, neurodegenerative diseases (Parkinson's, dementia), osteoarthritis (hand/knee/hip osteoarthritis, hip fracture). Depression was assessed using the Geriatric Depression Scale [14], and a score ≥11 was indicative of depressive symptoms. Cognitive function was assessed by administering the 30-item Mini Mental State Examination [15]. Scores for the MMSE range from 0 to 30, and a score below 24 is indicative of cognitive impairment [16]. Disability was defined as the inability or need for assistance to perform one or more activities of daily living (ADL): bathing, dressing, eating, using the toilet, or transferring.

Venous blood samples were obtained after an overnight fast, centrifuged and stored at −80°C. Routine biochemical tests were performed at city hospitals, and 25OHD tests at the Padua University laboratory. Serum 25OHD levels were measured by radioimmunoassay (RIA kit; Buhlmann Lab., Basel, Switzerland) in the ethanol extract of serum by a radiocompetitive assay using a binding protein obtained from rachitic rat serum. The sensitivity range is reported to be 1.25–160 ng/ml, and the coefficient of variation 5.3% and 8.9% intra- and inter-assay, respectively.

Serum intact PTH levels were measured using a two site immunoradiometric assay kit (N-tact PTHSP; DiaSorin): the intra-assay and inter-assay coefficients of variation for PTH were 3.0% and 5.5%, respectively. Serum creatinine was measured using a standard creatinine Jaffé method (Roche Diagnostics, Germany) and glomerular filtration rate (GFR) was calculated with the MDRD formula.

### Statistical analysis

Participants' characteristics were summarized using medians (first and third quartiles) for continuous variables, and counts and percentages for categorical variables. Medians and proportions were calculated for the following five age groups: 65–69 years, 70–74 years, 75–79 years, 80–84 years, ≥85 years. Given the known gender-related differences, all data analyses were stratified by sex. Differences in categorical variables were examined using the *chi*-square test, while the non-parametric Mann-Whitney test was used to check differences between medians of covariates by age group. In the whole sample and within each age group, the non-parametric test was also used to examine the differences in 25OHD median values by level of physical activity (dichotomized in ≥1 hour/week vs <1 hour/week).

Multivariate logistic regression models were used to examine the association between outdoor/indoor pastimes and the odds of vitamin D deficiency (defined as serum 25OHD levels <50 nmol/L). Known factors associated with 25OHD levels and/or physical functionality were examined for inclusion in the analyses and a multivariate model was obtained. Age, smoking habits (never/former vs current smoker), body mass index (BMI; calculated as the weight in kg/height in meters squared), season of the year (November–February vs March–October), cognitive impairment, depression, GFR, distance covered in the 6-minute walking test, CVD, neurodegenerative diseases, osteo-articular diseases, osteoporosis, cancer, diabetes, and COPD were all added as confounders in the model. The covariate-adjusted odds ratios (ORs) of vitamin D deficiency were obtained for each outdoor and indoor activity.

All analyses were performed using the SAS rel. 9.13 (Cary NC: SAS Institute). All statistical tests were two-tailed and statistical significance was assumed for a *p*-value <0.05.

## Results

### Participants' general characteristics

The sample consisted of 2,349 community-dwelling elderly subjects (960 men and 1,389 women). Their median age was 75 years (IQR 69–81.5) for the men and 74 (IQR 69–80) for the women. The median serum 25OHD levels were 95.0 nmol/L (IQR 61.6–133.5) in men and 59.0 nmol/L (IQR 38–88) in women. Vitamin D deficiency (25OHD <50 nmol/L) was more common among the females (34.2%), than among the males (11%); it was severe (25OHD <25 nmol/L) in 13% of the women and only 5% of the men. The proportions of participants regularly practicing at least one of the different outdoor and indoor leisure-time physical activities was generally higher for the men than for the women (84.1% vs 71.5%, p<0.0001, details not shown).

Tables 1 and 2 show the sample's general characteristics, as a whole and by age group, for men and women, respectively. In both genders, the median 25OHD levels tended to decline significantly from the youngest to the oldest individuals (p<0.0001). The proportions of subjects engaging in outdoor activities decreased with increasing age, with the exception of the brisk walkers, whose numbers did not drop significantly across age groups in the men (p = 0.26) or the women (p = 0.84). As for indoor activities, the proportion of men who danced and of women who attended the gym tended to decline (p = 0.14; p = 0.02, respectively).

**Table 1:** General characteristics in men (whole sample and by age group), expressed as medians (first and third quartiles) or percentages, as appropriate.

	Whole sample	Age group					p-value
		65–69 years	70–74 years	75–79 years	80–84 years	≥85 years	
	(n = 960)	(n = 262)	(n = 208)	(n = 184)	(n = 132)	(n = 174)	
Age (ys)	75.0 (69.0–81.5)	67.0 (66.0–68.0)	72.0 (71.0–73.0)	76.0 (75.0–78.0)	81.5 (81.0–83.0)	87.0 (86.0–89.0)	<0.0001
25OHD (nmol/L)	95 (61.5–133.5)	109.0 (77.0–144.0)	104.5 (77.0–142.0)	90.0 (60.0–139.5)	90.5 (56.5–121.27)	62.5 (46.0–99.0)	<0.0001
BMI (kg/m^2^)	26.67 (24.1–29.2)	27.76 (25.02–30.30)	27.37 (24.64–29.74)	26.01 (24.12–28.10)	25.96 (24.32–28.27)	25.34 (22.26–28.56)	<0.0001
6MWD (m)	369 (300–433)	424.0 (363.0–469.0)	392.0 (330.0–444.0)	377.0 (320.0–429.0)	329.0 (277.5–377.5)	264.0 (173.0–338.0)	<0.0001
COMORBIDITIES,%							
Depression	22.3	15.9	20.3	24.6	27.6	28.7	0.004
Cognitive impairment	33.2	12.6	22.7	31.5	43.5	71.3	<0.0001
Cardiovascular diseases	26.7	14.6	27.0	23.9	41.7	36.4	<0.0001
Neurodegenerative diseases	5.5	0.4	1.4	4.3	7.6	17.9	<0.0001
Osteo-articular diseases	26.2	19.1	27.7	26.1	28.2	33.5	0.001
Osteoporosis	24.0	15.6	14.4	25.5	29.5	41.9	<0.0001
Any cancer	8.7	6.9	6.2	10.9	7.6	13.2	0.47
Diabetes	8.0	9.1	7.2	10.3	7.6	5.2	0.27
COPD	15.2	10.3	12.5	12.5	18.2	26.4	<0.0001
OUTDOOR PHYSICAL ACTIVITY, %							
Brisk walking	39.7	41.5	33.5	45.5	38.3	39.3	0.26
Cycling	53.4	60.9	56.8	56.7	50.0	34.7	<0.0001
Gardening	45.7	52.3	49.5	52.2	40.6	25.3	<0.0001
Fishing	5.7	9.6	8.2	3.4	2.3	1.3	0.0008
INDOOR PHYSICAL ACTIVITY, %							
Dancing	3.5	5.4	3.9	3.9	1.6	0.7	0.14
Gym	7.0	10.4	7.8	4.5	3.9	6.0	0.26

25(OH)D: 25-hydroxy-vitamin D; ys: years; BMI: body mass index; COPD: chronic obstructive pulmonary disease; 6MWD: six-minute walking distance.

**Table 2:** General characteristics in women (whole sample and by age group), expressed as medians (first and third quartiles) or percentages, as appropriate.

	Whole sample	Age groups					p-value
		65–69 years	70–74 years	75–79 years	80–84 years	≥85 years	
	(n = 1389)	(n = 406)	(n = 325)	(n = 288)	(n = 210)	(n = 160)	
Age (ys)	74.0 (69.0–80.0)	67.0 (66.0–68.0)	72.0 (71.0–73.0)	76.0 (75.0–77.0)	82.0 (81.0–83.0)	87.0 (86.0–89.0)	<0.0001
25OHD (nmol/L)	59.0 (38.0–88.0)	71.0 (49.0–100.0)	66.0 (45.0–93.0)	59.0 (38.5–82.5)	47.5 (29.0–68.0)	37.5 (25.0–56.5)	<0.0001
BMI (kg/m^2^)	27.73 (24.87–30.70)	27.83 (25.19–31.03)	27.94 (25.10–30.87)	27.84 (24.90–30.52)	27.82 (24.54–30.77)	26.45 (23.32–29.56)	<0.0001
6MWD (m)	313.0 (240.0–373.0)	362.5 (313.0–408.0)	323.0 (273.0–380.0)	302.0 (240.0–360.0)	261.0 (180.0–320.0)	182.5 (124.5–249.0)	<0.0001
COMORBIDITIES,%							
Depression	38.5	32.7	32.8	42.5	43.4	53.7	<0.0001
Cognitive impairment	37.4	20.7	24.8	39.4	59.9	74.8	<0.0001
Cardiovascular diseases	17.3	6.9	13.0	19.4	26.7	36.2	<0.0001
Neurodegenerative diseases	5.5	0.2	1.5	4.2	8.6	25.0	<0.0001
Osteo-articular diseases	38.3	27.3	39.4	41.2	49.8	44.7	<0.0001
Osteoporosis	55.9	40.4	50.5	58.7	74.3	76.7	<0.0001
Any cancer	6.9	7.1	5.5	8.0	7.1	6.9	0.98
Diabetes	9.9	7.4	9.5	11.9	12.9	9.4	0.06
COPD	5.3	4.4	5.2	4.9	5.7	7.5	0.64
OUTDOOR PHYSICAL ACTIVITY, %							
Brisk walking	25.2	25.4	24.1	24.6	24.7	29.2	0.84
Cycling	35.8	51.2	46.6	32.0	14.8	3.6	<0.0001
Gardening	38.6	44.3	45.6	37.4	32.7	17.5	<0.0001
Fishing	-	-	-	-	-	-	-
INDOOR PHYSICAL ACTIVITY, %							
Dancing	0.9	1.2	0.9	1.1	0.5	0	0.54
Gym	8.3	12.2	8.1	6.4	6.4	4.4	0.02

25(OH)D: 25-hydroxy-vitamin D; ys: years; BMI: body mass index; COPD: chronic obstructive pulmonary disease; 6MWD: six-minute walking distance.

### 25OHD and physical activity

In both genders, median serum 25OHD level was higher in subjects who engaged in outdoor pastimes compared to those who did not (*for women:* 68 nmol/L (95% CI 64–71; IQR 45–94) vs 51 nmol/L (95% CI 48.54; IQR 32–74) respectively, p < 0.0001; *for men*: 101 nmol/L (95% CI 98–107; IQR 71–144) vs 73 nmol/L (95% CI 65–83; IQR 45–107) respectively, p < 0.0001).

Tables 3 and 4 show the median levels of 25OHD in individuals who did and did not practice the various outdoor and indoor activities by gender and age class. In all age groups, the men and women who regularly spent time gardening or cycling had significantly higher median 25OHD serum levels than those seen in participants who did not engage in these outdoor activities.

**Table 3:** Serum 25OHD levels (nmol/L) by age group and physical activity in men.

	Age groups				
	65–69 years	70–74 years	75–79 years	80–84 years	≥85 years
	(n = 262)	(n = 208)	(n = 184)	(n = 132)	(n = 174)
**OUTDOOR PHYSICAL ACTIVITY**					
Brisk walking <1 hour/week	105.5 (76.0–142.0)	106.0 (80.0–146.0)	99.0 (69.0–145.0)	95.0 (56.0–125.0)	65.0 (42.0–104.0)
Brisk walking >1 hour/week	110.0 (78.0–148.5)	104.0 (77.0–138.0)	89.0 (59.0–120.0)	89.0 (58.0–125.0)	57.0 (48.0–97.0)
Cycling <1 hour/week	102.0 (66.0–134.0)	99.0 (72.0–122.0)	81.0 (53.0–110.0)	75.5 (51.5–122.0)	55.0 (37.0–96.0)
Cycling >1 hour/week	111.0 (79.0–151.0) ^*^	114.0 (86.0–159.0) ^*^	105.0 (73.0–152.0) ^*^	96.5 (70.5–125.0) ^*^	82.5 (55.0–113.0) ^*^
Gardening <1 hour/week	103.5 (67.5–138.0)	99.0 (723.5–127.5)	86.0 (50.0–110.0)	89.0 (54.5–115.0)	57.0 (42.5–89.0)
Gardening >1 hour/week	110.5 (80.5–151.5) ^*^	112.5 (82.0–158.0) ^**^	110.0 (73.0–156.0) ^**^	98.0 (70.0–134.5) ^*^	98.5 (50.0–148.0) ^**^
Fishing <1 hour/week	106.0 (76.0–142.0)	104.0 (77.0–136.0)	90.5 (60.0–144.0)	92.0 (57.0–125.0)	60.0 (45,5–99.5)
Fishing >1 hour/week	116.0 (96.0–159.0) ^*^	150.0 (94.0–186.0) ^*^	116.0 (79.0–136.0)	75.0 (64.0–108.0)	89.0 (12.0–166.0)
**INDOOR PHYSICAL ACTIVITY**					
Dancing <1 hour/week	109.5 (77.0–144.0)	102.5 (77.0–138.0)	90.0 (59.0–144.0)	91.5 (57.0–125.0)	60 (45.0–100.0)
Dancing >1 hour/week	98.5 (53.0–137.0)	139.0 (119.0–208.0) ^*^	120.0 (80.0–136.0)	84.0 (70.0–98.0)	79.0
Gym <1 hour/week	108.0 (7.0–140.0)	104.0 (77.0–146.0)	90.5 (60.0–144.0)	92.0 (57.0–128.0)	62.0 (46.0–100.0)
Gym >1 hour/week	145.0 (70.0–160.0)	124.0 (82.0–140.5)	93.5 (62.0–151.0)	70.0 (51.0–91.0)	51.0 (35.0–73.0)

***Comparison between age groups***: ^*^ p<0.05; ^**^ p<0.01.

**Table 4:** Serum 25OHD levels (nmol/L) by age group and physical activity in women.

	Age groups				
	65–69 years	70–74 years	75–79 years	80–84 years	≥85 years
	(n = 406)	(n = 325)	(n = 288)	(n = 210)	(n = 160)
**OUTDOOR PHYSICAL ACTIVITY**					
Brisk walking <1 hour/week	71.0 (49.0–100.0)	68.0 (45.0–93.0)	57.0 (37.0–79.0)	46.5 (31.5–67.5)	40.0 (28.0–57.0)
Brisk walking >1 hour/week	73.0 (50.0–99.0)	65.0 (40.0–91.0)	66.0 (40.0–92.0) ^*^	53.0 (28.0–73.0)	36.0 (30.0–71.5)
Cycling <1 hour/week	66.0 (44.0–94.5)	62.0 (40.0–86.0)	55.0 (37.0–79.0)	46.0 (28.0–65.5)	38.0 (28.0–57.0)
Cycling >1 hour/week	77.0 (54.0–105.0) ^*^	76.0 (52.0–102.0) ^**^	65.5 (48.0–88.0) ^**^	58.5 (42.0–90.0) ^*^	37.0 (35.0–72.0)
Gardening <1 hour/week	68.0 (46.0–98.0)	62.0 (41.0–88.0)	55.0 (37.0–80.5)	46.0 (28.0–65.5)	37.0 (25.0–56.0)
Gardening >1 hour/week	76.0 (53.0–103.0) ^*^	73.0 (47.0–97.0) ^*^	61.0 (45.0–85.0) ^*^	53.0 (33.0–82.0) ^**^	45.5 (35.5–83.0) ^**^
**INDOOR PHYSICAL ACTIVITY**					
Dancing <1 hour/week	71.0 (49.0–100.0)	66.0 (45.0–93.0)	58.5 (38.0–83.0)	50.0 (31.0–69.0)	–
Dancing >1 hour/week	80.0 (66.0–139.0)	72.0 (43.0–84–0)	65.0 (60.0–88.0)	10.0	–
Gym <1 hour/week	70.0 (48.0–98.0)	66.0 (45.0–92.0)	59.0 (40.0–82.0)	47.0 (30.0–69.0)	38.0 (28.0–57.0)
Gym >1 hour/week	86.0 (65.0–120.0) ^**^	76.5 (51.0–115.0)	40.0 (30.0–89.0)	57.0 (36.0–66.0)	35.0 (22.0–71.0)

***Comparison between age groups***: ^*^ p<0.05; ^**^ p<0.01.

The median 25OHD levels did not differ significantly between men who did or did not walk regularly, whereas for the women the difference in 25OHD serum levels was significant only for those aged 75–79. Men aged 65–75 years who went fishing regularly had significantly higher serum 25OHD levels than those who did not; none of the women engaged regularly in this activity.

Among the indoor pastimes considered, individuals who danced or attended the gym did not have significantly higher 25OHD levels than those who did not, except for men aged 70–74 years who danced regularly, and 65- to 74-year-old women who exercised at the gym. The small numbers of participants engaging in indoor activities (especially after splitting them into the five age groups considered) may have negatively affected the likelihood of a significant difference coming to light, however.

**Figure 1:**
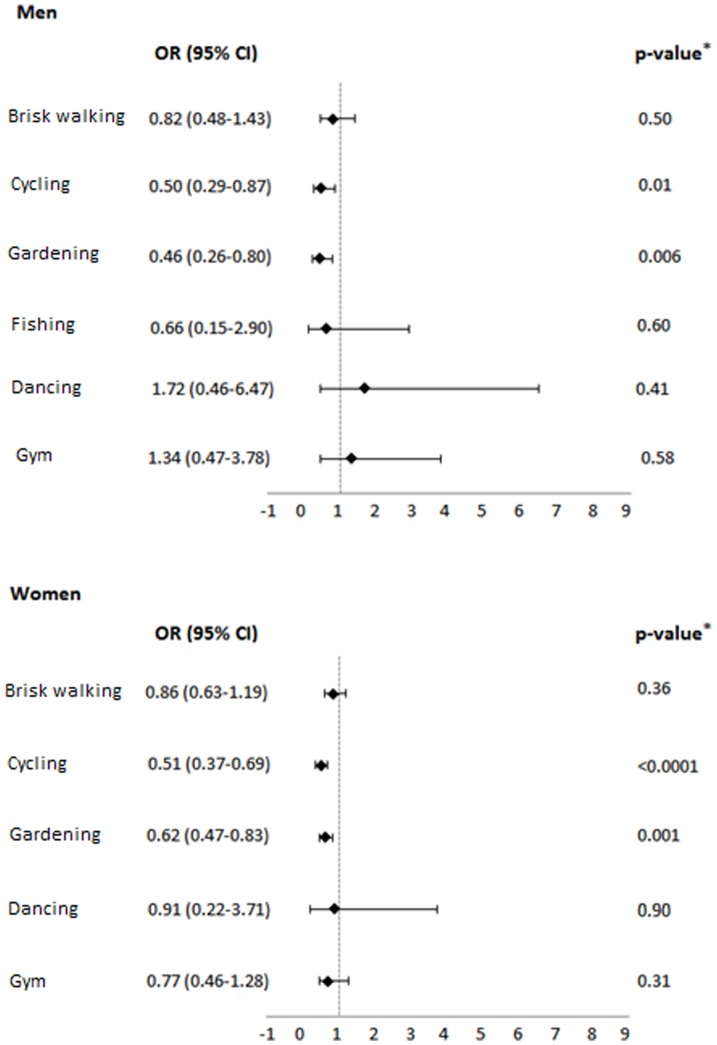
Results of logistic regression analysis for significant independent factors associated with vitamin D deficiency. ^*^ Adjusted for age, body mass index, smoking habits, season, 6-minute walking distance, cognitive status, depression, cardiovascular diseases, neurodegenerative diseases, osteo-articular diseases, osteoporosis, cancer, diabetes, and chronic obstructive pulmonary disease.

The covariate-adjusted odds ratios of vitamin D deficiency (defined as serum 25OHD levels <50 nmol/L) for each of the outdoor and indoor activities are shown in Figure 1. The indoor pastimes showed no significant association with low 25OHD levels. Among the outdoor activities, both men and women who were regularly cycling or gardening were less likely to be deficient in vitamin D (*for cycling*: OR 0.50, 95% CI 0.29–0.87 in men, and OR 0.51, 95% CI 0.37–0.69 in women; *for gardening*: OR 0.46, 95% CI 0.26–0.80 in men, and OR 0.62, 95% CI 0.47–0.83 in women). On the other hand, brisk walking and fishing were unassociated with any lower likelihood of hypovitaminosis D in either gender.

## Discussion

The present study on an ample sample of Caucasian elderly subjects living in north-east Italy (45°N) showed that different outdoor activities might not have the same effects in maintaining high vitamin D levels. Our findings suggest that outdoor pastimes involving a considerable exposure to sunlight, such as cycling and gardening - but not brisk walking - are associated with a lower probability of vitamin D deficiency in well-performing, community-dwelling elderly men and women.

In our sample, serum 25OHD concentrations were generally higher in men than in women, whose median level (59.0 nmol/L) was lower than the men's 25^th^ percentile (61.5 nmol/L). This finding is consistent with a study by Maeda et al. [17], who analyzed 25OHD levels in three groups of elderly patients (nursing-home residents and community-dwelling elderly who did or did not get regular exercise), finding serum vitamin D concentrations higher in men than in women in all three groups. This might be because men have traditionally engaged more in outdoor activities, as confirmed by the higher proportion of men than women in our sample practicing some sort of physical activity. As van Dam et al. [18] pointed out, however, women's higher percentage of fat mass compared to men [19] may increase the former's vitamin D storage in adipose tissue and account for females' lower serum 25OHD concentrations.

Our findings indicate that serum 25OHD levels gradually decrease with age in both genders. So do people's tendencies to engage in outdoor physical activities as they get older, although this cannot be explained by physical disability (as reported in the Methods, participants unable to complete the standardized 6MWT were excluded and only well-performing subjects with no disabilities were considered in the present study). This picture might be due to some activities (e.g. gardening, cycling, dancing, fishing) requiring more strength, coordination and balance than is needed for brisk walking (an activity for which the participation rates remained fairly stable across age groups in both genders).

Concerning the relationship between physical activity and 25OHD levels, as was to be expected, we found no association between indoor activities and hypovitaminosis D. Although it would seem obvious that indoor physical activity does not modify 25OHD levels, some authors have suggested that physical activity per se may raise vitamin D levels [20], [21], possibly by means of a transient exercise-induced increase in PTH levels [22].

It is currently assumed that any outdoor activity exposing people to more or less sunlight should improve vitamin D status, but our findings suggest that this is not entirely so. Among the outdoor pastimes considered in our large population-based sample, only cycling and gardening (not brisk walking or fishing) were associated with a lower likelihood of having serum 25OHD concentrations <50 nmol/L. In the large cohort of elderly subjects from the National Health and Nutrition Examination Survey (NHANES) III, outdoor activities (walking, jogging and gardening) were found associated with higher levels of serum 25OHD; unfortunately these three activities were clustered together and no details were reported on the association between each of these activities and 25OHD serum levels [10]. There may be several reasons why not all outdoor activities have the same association with serum vitamin D levels. For instance, gardening is rarely only an occasional pastime, it usually demands a certain regularity, and it can keep someone occupied for hours on a daily basis. People who are gardening are also quite likely to wear light clothes and expose a greater body surface area to sunlight. The same can be said of cyclists, whereas brisk walkers may well be wearing more clothes and engaging in this activity early in the morning or in the evenings, when the UV radiation from the sun is feeble. Unfortunately the time of day when participants engaged in their pastimes was not recorded, and this is the main shortcoming of our study. Another limit of the present study lies in that the activities and the number of hours spent per week in the previous month were self-reported by participants during face-to face interviews, so the chances of an under- or over-reporting bias should be taken into account.

The main strengths of our study lie in its population-based design and large sample size, comprising a proportion of men and women representative of the general elderly population of north-east Italy. Another strength relates to the large number of confounders and diagnosed diseases investigated. The proportion of participants taking vitamin D supplements was less than 1% in our study sample as a whole, so the serum 25OHD levels identified in our study were hardly influenced by its oral supplementation. In addition, all participants in the present study were well-performing and in good health, as demonstrated by the results of the standardized 6-minute walking test, which is an indirect marker of aerobic capacity and exercise tolerance [13].

In conclusion, the present study demonstrates that outdoor physical activities are not all equally beneficial in terms of vitamin D status. Engaging for at least an hour a week in activities such as cycling or gardening seems to help reduce the likelihood of vitamin D deficiency in well-performing elderly people. Considering all the extraskeletal effects of vitamin D, spending time regularly in these activities (even only for a short time each week) could have other overall health benefits, as well as a positive effect on cardiovascular performance. Campaigns aiming to contain the hypovitaminosis D *epidemic* among older adults should not only recommend vitamin D supplementation, but also promote the benefits of this kind of outdoor pastime.
